# Expression analysis of calmodulin and calmodulin-like genes from rice, *Oryza sativa* L.

**DOI:** 10.1186/1756-0500-5-625

**Published:** 2012-11-08

**Authors:** Aumnart Chinpongpanich, Kampon Limruengroj, Srivilai Phean-o-pas, Tipaporn Limpaseni, Teerapong Buaboocha

**Affiliations:** 1 Department of Biochemistry, Faculty of Science, Chulalongkorn University, Bangkok, 10330, Thailand

**Keywords:** Calcium signaling, Calmodulin, CaM, CML, Rice, *Oryza sativa*

## Abstract

**Background:**

In plants, a large family of calmodulin (CaM) and CaM-like (CML) proteins transduce the increase in cytosolic Ca^2+^ concentrations by binding to and altering the activities of target proteins, and thereby affecting the physiological responses to a vast array of stimuli. Here, transcript expression analysis of *Cam* and *CML* gene family members in rice (*Oryza sativa* L.) was extensively examined.

**Results:**

*Cam* and *CML* genes in rice exhibited differential expression patterns in tissues/organs. Under osmotic stress and salt stress, expression of *OsCam1-1*, *OsCML4*, *5*, *8*, and *11* was induced with different kinetics and magnitude. *OsCML4* and *8* mRNA levels significantly increased by 3 h after treatment and remained elevated for at least 24 h while expression of *OsCam1-1*, *OsCML5* and *11* was up-regulated as early as 1–3 h before rapidly returning to normal levels. Several *cis*-acting elements in response to abiotic stresses, including DREs (important promoter elements responsive to drought, high salt, and cold stress), were detected in the 5^′^ upstream regions of these genes. The observed induction of the GUS activity of transgenic rice plants via the *OsCam1-1* promoter appeared to be biphasic and dependent on the severity of salt stress.

**Conclusions:**

Large *OsCam* and *OsCML* gene family members likely play differential roles as signal transducers in regulating various developmental processes and represent important nodes in the signal transduction and transcriptional regulation networks in abiotic stresss responses mediated by the complex Ca^2+^ signals in plants, which are rich in both spatial and temporal information.

## Background

Transient changes in the cytosolic Ca^2+^ concentration ([Ca^2+^_cyt_) of a different magnitude and specialized character due to the activities of Ca^2+^-ATPases and Ca^2+^-channels in the cellular membrane of eukaryotes are utilized as a second messenger in generating physiological responses to extracellular stimuli. The use of Ca^2+^ signals has been implicated in generating responses to a wide variety of environmental changes in plants [1]. The changes in [Ca^2+^_cyt_ are not only transient, but also vary spatially and temporally with different organelles acting as distinct compartments [2], therefore, the diverse array of different changes in the [Ca^2+^_cyt_ must be discriminated so as to elicit the correct subsequent cellular response, a task performed by various Ca^2+^-modulated proteins. For the majority of these proteins, the Ca^2+^-binding sites are composed of a characteristic helix-loop-helix motif called an EF-hand [3], which binds Ca^2+^ with high affinity, resulting in conformation changes that modulate their activity or their ability to interact with other proteins.

Plants possess a complex network of Ca^2+^ signal transduction in mediating responses to various biotic and abiotic environmental stimuli. In plants, genes encoding EF-hand containing proteins have been extensively annotated in *Arabidopsis thaliana* (L.) Heynh [4] and rice (*Oryza sativa* L.) [5]. Three groups of EF-hand-containing Ca^2+^ sensor proteins have been identified, which include Ca^2+^-dependent protein kinase (CPK), calcineurin B-like protein (CBL), and calmodulin (CaM) [6]. These proteins can be considered as sensor responders or sensor relays [7]. CPKs, and CBLs together with their respective CBL-interacting protein kinases (CIPKs), are sensor responders as they possess kinase activity either in their molecules or in their high affinity interacting partners as a responder function. By contrast, CaMs and CMLs, which have no other identifiable functional domain other than EF-hand motifs, are considered sensor relays. A large family of *Cam* and *Cam-like* (*CML*) genes has been extensively identified from the two model plants, *A. thaliana*[8] and *O. sativa*[5]. Although the existence of these proteins in a single plant species is believed to be important to correctly perceive and discriminate the Ca^2+^ signals from different stimuli, and thus aid in eliciting the correct subsequent response, the molecular mechanisms and physiological significance of most of these proteins have not been established. Nevertheless, accumulating evidence suggests that each of the different *Cam* and *CML* genes may have distinct and significant functions [9].

Currently, the information on *Cam* and *CML* genes in *O. sativa* is quite limited despite the fact it is a model plant for monocot and especially cereal plants. Regarding expression studies, reports on only a few members of the *OsCam* and *OsCML* gene family, are available [10,11]. Here, the transcript expression levels of several members of the *OsCam* and *OsCML* gene family were examined in *O. sativa* that had experienced abiotic stresses. Their 5^′^ upstream sequences were examined to identify putative stress-responsive *cis*-acting elements. The putative promoter of the *OsCam1-1* gene was further examined using three transgenic rice lines. Based on their expression patterns, the *OsCam* and *OsCML* genes that are likely to represent important nodes in the signal transduction and transcriptional regulation networks of Ca^2+^ signaling in response to different abiotic stresses will be revealed, and subject to confirmation, this is likely facilitate the efforts in characterizing the mechanisms of abiotic stress Ca^2+^ signalling in plants.

## Results

### Differential expression of *OsCam* and *OsCML* gene family members in various organs

**Figure 1:**
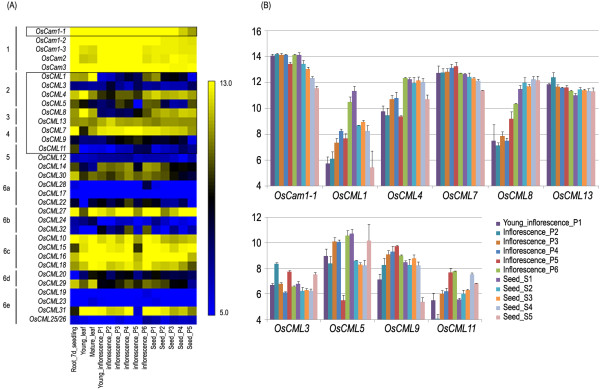
***OsCam *and *OsCML *gene expression in various organs and developmental stages based on DNA microarray.(A)** Expression profiles of the *Cam* and *CML* genes in the *O. sativa* IR64 cultivar shown as a heat map. The groupings designated as 1–6 based on the phylogenetic analysis of CaM and CML proteins are indicated on the left and the colour scale is shown on the right. **(B)** and **(C)** Expression profiles of *OsCam1-1* and the nine *CML* genes shown as bar graphs. Data in **(B)** and **(C)** are shown as the mean ± 1 SD, and are derived from three independent replicates.

The rice (*Oryza sativa* L.) genome has been reported to encode for a large family of five *Cam* genes and 32 genes encoding CML calcium sensor proteins [5]. To determine which of these *OsCam* and *OsCML* genes may have tissue- and/or stress- specific expression patterns, which could suggest their functions, their expression levels in different rice tissues and organs were investigated using publicly available microarray data of global gene expression from the experiment GSE 6893 from the *O.sativa* IR64 cultivar [12]. The available expression data of all *OsCam* and *OsCML* genes plus *OsEF1α* were retrieved and the results are shown as heat map in Figure 1A, and are grouped according to the previously reported clades found by phylogenetic analysis of their amino acid sequences. It is clearly noticeable that all the *OsCam* genes (group 1) are highly expressed in almost all of the organs examined. In contrast, several highly conserved *OsCML* (groups 2–5) genes tend to be highly expressed in some organs/tissues. Interestingly, one member of each subgroup in group 6 is found expressed at a higher level compared with the other members in their subgroup (*OsCML30* in group 6a; *OsCML27* in group 6b; *OsCML29* in group 6c; *OsCML31* in group 6e) except group 6C, in which all members are highly expressed in most organs examined.

Transcript expression levels of ten genes, one *Cam* (*OsCam1-1*) and nine highly conserved *CML* genes (Os*CML1*, *3*, *4* and *5* in group 2, *OsCML8* and *13* in group 3, *OsCML7* and *9* in group 4, and *OsCML11* in group 5) were closely examined in different rice organs and tissues (Figure 1B). Our preliminary results indicated that *OsCam1-1* gene is the only *OsCam* gene which expression is induced by osmotic stress and salt stress, therefore, only *OsCam1-1* among the five *OsCam* genes was selected for this study. Based on the microarray data analysis, ubiquitous transcript expression at relatively high levels of *OsCam1-1*, *OsCML7* and *13* was observed. The other three genes, *OsCML1*, *4*, and *8* had similar expression patterns with higher levels in the leaf and the root and lower levels in the young inflorescence (Figure 1A). Their expression levels were then increasing upon maturation of the inflorescence until reaching similar levels to those of the leaf and the root in the early developing seed. Expression levels of *OsCML4*, and *8* were maintained in the seed until dormancy and desiccation while that of *OsCML1* later decreased upon seed maturation (Figure 1B). For the other four genes: *OsCML3*, *5*, *9*, and *11*, overall, they exhibited relatively lower expression levels compared to the aforementioned genes.

To examine whether these genes are expressed in the three-week old Khao Dawk Mali 105 (KDML105) rice cultivar seedlings and to optimize the PCR conditions, RT-PCR was performed using the oligonucleotide primers specific for each gene (Table 1). Following separation of the PCR products in agarose-TBE gels and visualization by uv-transillumination after ethidium-bromide staining, bands of the expected size based on the *OsCam1-1* and Os*CML* sequences (Table 1) were specifically detected (data not shown). Thus, transcripts of *OsCam1-1* and all nine *OsCML* genes examined are expressed in leaves of the three-week old ‘KDML105’ rice seedlings, and can be specifically amplified by RT-PCR under these conditions.

**Table 1:** Oligonucleotide primers used in this study

**Primer name**	**Sequence**	**Position**^**a**^	**Amplicon size (bp)**^**b**^	**Annealing temp. (°C)**
*OsCam1-1*-F	5^′^- ACCGTGCATTGCCGTATTAG -3^′^	499-518	177	58.3
*OsCam1-1*-R	5^′^- GCAAGCCTTAACAGATTCAC -3^′^	656-675		
*OsCML1*-F	5^′^- CCAGAAGTGCGTGATCCTGT -3^′^	543-562	184	58.3
*OsCML1*-R	5^′^- ACTACGGACTACGGCTGTGA -3^′^	707-726		
*OsCML3*-F	5^′^- ACTACAACGAGTTCCTCAAG -3^′^	410-429	180	57.3
*OsCML3*-R	5^′^- CATCAGAACAGTTGCAAACC -3^′^	570-589		
*OsCML4*-F	5^′^- GCAGGTGAACTACGATGAAT -3^′^	402-421	193	56.3
*OsCML4*-R	5^′^- TACCCATAGCTGAAGTCCAA -3^′^	575-594		
*OsCML5*-F	5^′^- ATGATGCTCTCCGACCAATA -3^′^	481-500	180	57.3
*OsCML5*-R	5^′^- CCAAGGCCAAATTAAATGAC -3^′^	641-660		
*OsCML7*-F	5^′^- CCGCATCGTCGCCAAATAAT -3^′^	429-448	193	57.3
*OsCML7*-R	5^′^- GTCCAAATCACACCGGAATG -3^′^	602-621		
*OsCML8*-F	5^′^- AGATGATGAAGAGGATAGGA -3^′^	539-555	185	56.3
*OsCML8*-R	5^′^- AAACATAAGGCGGTATGGTA -3^′^	701-720		
*OsCML9*-F	5^′^- TACAAGGAGTTCGTCAAGGT -3^′^	430-449	170	58.3
*OsCML9*-R	5^′^- GATTCGCTTGAATCATATCGC -3^′^	579-600		
*OsCML11*-F	5^′^- CAACATCTTCTCCTGAGAAT -3^′^	621-640	183	56.3
*OsCML11*-R	5^′^- ATTCACAAGAGCTCGATCAC -3^′^	784-803		
*OsCML13*-F	5^′^- ATCGAAATGGTGATGGTGAG -3^′^	437-456	193	58.3
*OsCML13*-R	5^′^- GCATGGTTGTTCTTGTTCAG -3^′^	610-629		
*OsEF1α-*F	5^′^- ATGGTTGTGGAGACCTTC -3^′^	1192-1209	127	58.3
*OsEF1α-*R	5^′^- TCACCTTGGCACCGGTTG -3^′^	1301-1318		

^a^Position of the primers from the GenBank sequence given in the materials and methods, where position 1 is the predicted open reading frame start codon and numbered 5^′^ to 3^′^ on the sense strand.

^b^Expected amplicon size based upon the primer positions on the GenBank sequence.

**Figure 2:**
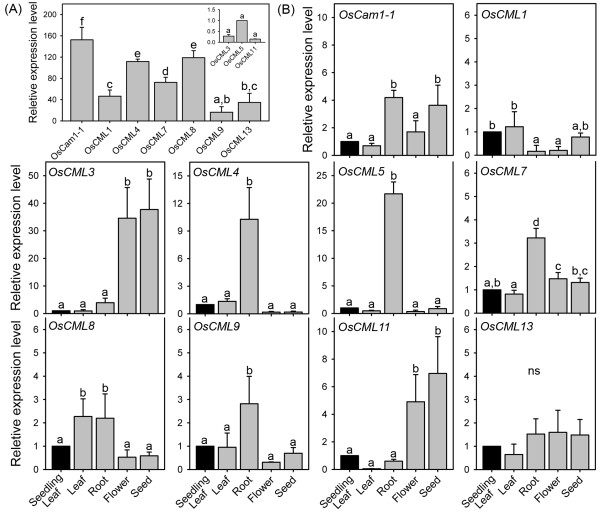
***OsCam *and *OsCML *transcript expression levels in various ‘KDML105’ rice organs as determined by rt-RT-PCR.** Relative transcript expression levels of *OsCam1-1* and the nine *OsCML* genes in **(A)** leaf blades/sheaths of three-week old ‘KDML105’ rice seedlings relative to *OsCML5* and in **(B)** leaf blades/sheaths, roots, flowers, and seeds of the ‘KDML105’ rice during the grain filling period compared with their respective level in the leaf of the three-week old rice seedlings. Data are shown as the mean ± 1 SD, and are derived from three independent replicates. Means with a different lowercase letter are significantly different (p < 0.05).

The transcript expression levels of these genes in the leaf blades/sheaths of the three-week old ‘KDML105’ rice seedlings, was evaluated by rt-RT-PCR using the transcript expression level of *OsCML5* as reference (Figure 2), *OsCam1-1* was found to have the highest expression level (~150-fold higher than *OsCML5*) in the leaf tissue of the three-week old rice seedlings, followed by *OsCML4*, *8* (~120-fold) (Figure 2A). *OsCML1*, *7*, and *13* also exhibited a relatively high expression level (>30-fold), while *OsCML3*, *5*, *9*, and *11* expressed at a relatively lower level.

The transcript expression levels of *OsCam1-1* and all nine O*sCML* genes assayed in the leaf blades/sheaths, roots, flowers, and seeds of the KDML105 rice cultivar during the grain filling period were determined and compared with their respective levels in the leaf tissue of the three-week old rice seedlings (Figure 2B). The expression profiles of these genes may be divided into three groups when only large differences (≥5-fold) among organs were considered. The first group, which has the highest expression levels in the root includes *OsCML4*, and *5*. The second group with the highest expression levels in the flower and the seed were *OsCML3*, and *11*. Finally, *OsCam1-1* and the five other *OsCML* genes (*OsCML1*, *7*, *8*, *9*, and *13*) had more or less similar expression levels among these organs and comprised the third group.

### Expression of *OsCam* and *OsCML* genes under abiotic stress

To investigate whether *OsCam1-1* and the nine selected *OsCML* genes may possibly be involved in mediating responses to abiotic stress, the publicly available rice microarray data was retrieved and examined to acquire data for the IR64 rice cultivar, whilst the transcript and expression levels in the ‘KDML105’ rice were evaluated after different abiotic stresses by rt-RT-PCR.

**Figure 3:**
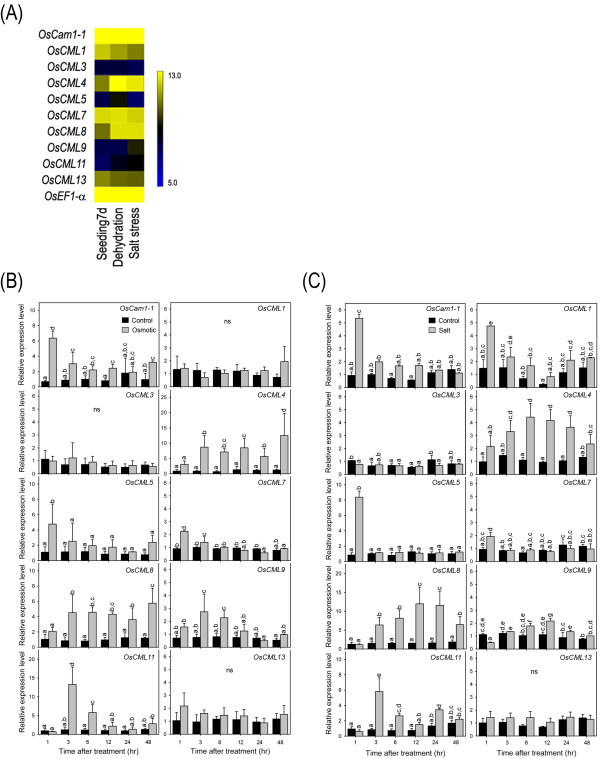
***OsCam *and *OsCML *transcript expression in response to stress.(A)** Microarray based transcript expression profiles under dehydration (drought) and salt stress shown as a heat map. Transcript expression levels examined in the KDML105 rice cultivar under osmotic stress **(B)** and salt stress **(C)** showing relative transcript expression levels standardized to that of *OsEF1α* and expressed relative to the levels on day 0 of the treatment. Rice seedlings were grown in a CRD with three replicates, and for each replicate, ten seedlings were pooled for RNA extraction. The PCR reaction of the same cDNA preparation was performed in triplicate for technical replication. Data are shown as the mean ± 1 SD and means with a different lowercase letter are significantly different (p < 0.05).

The results of the DNA microarray data of *OsCam1-1* and the nine *OsCML* genes in the 7-day-old ‘IR64’ rice seedlings grown under 3 h of dehydration (drought) or salt stress (200 mM NaCl) (GSE 6901) [12] retrieved via the rice oligonucleotide array database are shown as a heat map in Figure 3. The transcript expression levels of *OsCam1-1*, and especially of *OsCML4*, *5*, *8*, and *11* were significantly increased under dehydration, whilst that of *OsCML13* was slightly down-regulated. Under salt stress, significantly increased transcript expression levels of *OsCam1-1*, and especially of *OsCML4*, *8*, *9*, and *11* were observed, whilst that for *OsCML13* was slightly down-regulated. Note that although numerically the transcript expression level of *OsCML3* was increased following both dehydration and salt stress, this was not statistically significant due to the large variance in the control samples, and so remains to be clarified.

With respect to the three-week-old *O.sativa* ‘KDML105’ seedlings, rt-RT-PCR revealed that under osmotic stress (20% PEG), *OsCML4* and *OsCML8* exhibited a nine- and four- fold increases in their transcript expression levels, respectively, and that this started from 3 h after osmotic stress and were maintained at these elevated expression levels until at least 48 h after treatment (Figure 3B). However, whilst significantly elevated transcript expression levels of *OsCML11* were also detected at 3 h after osmotic stress (13-fold), in contrast these expression levels then declined to six-fold higher and control levels at 6 h and 12 h onwards after osmotic stress, respectively. Expression of *OsCam1-1,* and *OsCML5* showed an earlier response, being up-regulated 5-6-fold at 1 h after osmotic stress treatment compared with the control, but whilst *OsCam1-1* expression levels remained slightly up-regulated at 3 to 48 h after osmotic stress that for *OsCML5* returned to their normal levels after 3 to 6 h. *OsCML7* and *OsCML9* exhibited a slight increase in their transcript expression level at early time points (at 1–3 and 3–6 h after treatment, respectively), while no significant changes in the transcript expression levels of *OsCML1*, *3* and *13* were observed at all time points examined.

Similar to osmotic stress, *OsCML4* and *OsCML8* also showed significant prolonged increases in their transcript expression levels under salt stress (150 mM NaCl), starting from 3 h until at least 24 h after treatment (Figure 3C). Compared to the respective control, the *OsCML4* transcript expression levels were maximal at a four-fold elevated level at 6 and 12 h after treatment while *OsCML8* reached the highest levels (12-fold) at 12 and 24 h after treatment. Early up-regulated transcript expression of *OsCam1-1* and *OsCML5* at 1 h after treatment (five- and eight-fold, respectively) and of *OsCML11* at 3 h after treatment (six-fold) was also observed. In contrast, the transcript expression levels of *OsCML3* and *OsCML13* were not significantly altered in response to salt stress at all time points examined in this study, whilst *OsCML7* and *OsCML9* were only slightly and transiently increased at 1 h and 6 to 12 h after salt stress, respectively. However, unlike under osmotic stress, *OsCML1* exhibited increase in its expression at 1 h after salt stress.

### Examination of the *OsCam1-1* and *OsCML* promoters

**Figure 4:**
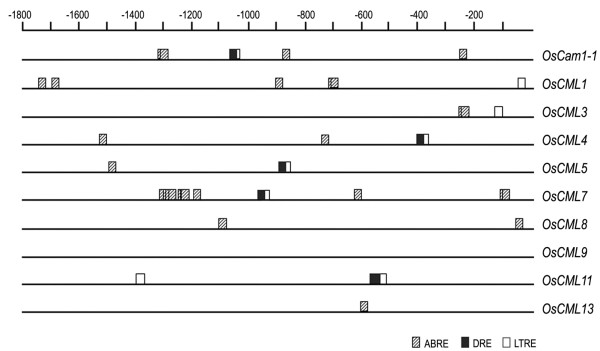
***Cis*-acting elements involved in response to abiotic stress in the *OsCam1-1 *and *OsCML *promoters.** The 5^′^ upstream regions are represented by lines and the putative ABRE, DRE and LTRE sequences are shown with a striped rectangle, a close rectangle, and an open rectangle, respectively.

In order to examine the promoters of these genes and their *cis*-acting elements, the sequences upstream of their coding regions were retrieved from GenBank and bioinformatically analyzed using the PLACE [13,14] and the PlantPAN [15] software and dtabases. Several characteristic elements were located; including motifs involved in responses to dehydration, abscisic acid (ABA), salt stress, and cold stress (Figure 4). Putative DREs (drought-responsive elements) [16] were found in the 5^′^ flanking (upstream) region of five of the loci (*OsCam1-1* at −1064, *OsCML4* at −393, *OsCML5* at-892, *OsCML7* at-954, and *OsCML11* at-561 and −565). In addition, the 5^′^ upstream sequences from eight of these genes (*OsCam1-1*, *OsCML1*, *3*, *4, 5, 7*, *8* and *13*) contained putative ABREs (ABA-responsive elements) [17], while LTREs (low temperature responsive elements) [18] were found in seven of these genes (*OsCam1-1*, *OsCML1*, *3*, *4*, *5, 7*, and *11*).

**Figure 5:**
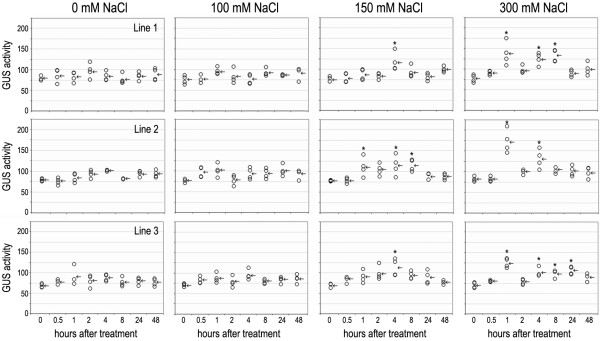
**GUS activity of the three transgenic rice lines over-expressing *OsCam1-1*:*gus *under salt stress.** Each circle and arrow indicates each value of GUS activity (units per μg protein) and the mean of those values, respectively. The asterisks indicate significantly higher GUS activity compared with their respective control (p < 0.05).

To investigate inducibility of the *OsCam1-1* promoter by salt-stress, (0 (control), 100, 150 or 300 mM NaCl), the β-glucuronidase (GUS) activity of three independent lines of the homozygous T3 transgenic KDML105 rice cultivars harbouring the *OsCam1-1::gus* construct was examined fluorometrically in two-week-old seedlings. All three independent transgenic lines exhibited similar trends of GUS activity to each other under the different NaCl treatments (Figure 5). The plants treated with 100 mM NaCl did not exhibit any statistically significant difference in GUS activity in all three independent lines (p < 0.05) compared to their respective controls at 0 and the untreated plants, whereas at 150 mM NaCl all transgenic lines had exhibited a significantly higher GUS activity from 4 h after treatment in lines 1 and 3, and after 1 to 8 h in line 2. For the plants treated with 300 mM NaCl, a biphasic induction of GUS activity was observed in all three independent transgenic lines with an initial induction and highest induction of GUS activity at 1 h after treatment, which then decreased to the control level by 2 h and was followed by the second phase of induction at 4 h after treatment. This second phase was either transient (line 2) or maintained for up to 8 h (line 1) or 24 h (line 3) after treatment.

## Discussion

Whilst the OsCaM group contains five members that share a highest degree of amino acid sequence identity (≥ 97%) to known typical CaMs from other plants, previous report phylogenetically classified the highly conserved OsCML proteins into four groups [5]. The OsCML proteins are small proteins of 145 to 250 amino acid residues with an approximately 44% to 85% amino acid identity to typical plant CaMs. A large family of six *Cam* and 50 *CML* genes have also been annotated in the *A.thaliana* genome [8], suggesting the existence of an extensive set of CaM and CML proteins in each plant species.

Even though rice is considered an important crop and a model for other monocots and especially cereal crops, almost all of *OsCam* and *OsCML* genes have not been characterized. From the publicly available microarray data, all *OsCam* genes were found highly expressed in almost all organs examined (Figure 1A). One of the defining characteristics of CaMs in plants is the presence of multiple CaM isoforms. Even though they are ubiquitously expressed, the different isoforms can display differential responses to individual stimuli in any given plant tissue [5,11,19-22], suggesting that each of the *OsCam* genes may have distinct physiological functions depending on where and how the expression of each gene is up-regulated in addition to the different biochemical properties that might be affected by the slight differences in their primary structures [21,23].

In contrast, several highly conserved *OsCML* genes (groups 2–5) genes tend to be highly expressed in some organs/tissues and their levels of expression are modulated during different stages of development (Figure 1A), indicating that they are developmentally regulated. It is conceivable that CaM proteins have many targets and are ubiquitously involved in numerous cellular processes while the highly conserved CML proteins have more specialized targets and are involved in more specific processes of the cell. Interestingly, each subgroup of the highly diverged group of *OsCML* (group 6a to 6e) genes had one member whose expression is found at a noticeably higher level than the other members. Members in the same subgroups have the same number and configuration of EF-hand motifs. It is speculated that these highly expressed genes may be the predominant gene representing each subgroup that has essential functions in the cell.

When the microarray data was closely examined for the transcript expression levels of the *OsCam1-1* and nine *OsCML* genes (Figure 1B), ubiquitous expression at relatively high levels of *OsCam1-1*, *OsCML7*, and *13* was observed, which suggests that they may have important functions during the regular growth and development processes of rice plants. The increasing transcript expression levels of *OsCML1*, *4*, and *8* with maturation of the inflorescence until they reached similar levels as those in the leaf and root tissues in the early developing seed, suggest that they may have important roles during the maturation of the inflorescence and the early seed development, especially *OsCML1*, which expression level peaked during those stages.

The transcript expression levels in the ‘KDML105’ rice in the leaf blades/sheaths of the three-week old ‘KDML105’ rice seedlings, as evaluated by rt-RT-PCR (Figure 2A) agree well with the microarray data in the IR64 cultivar (Figure 1A). When the transcript expression levels of *OsCam1-1* and *OsCML*s were examined in different organs (Figure 2B), their profiles could be divided into three groups as those with the highest expression level in (i) the root, or (ii) in the flower and the seed, or (iii) those genes with similar expression levels among the organs examined. Relatively high transcript levels of *OsCML3* and *OsCML11* in the flower and the seed, and *OsCML4*, and *5* in the root suggest their functional significance in those respective organs. The *OsCam1-1* and the other five *OsCML* genes (*OsCML1*, *7*, *8*, *9* and *13*) had more or less similar expression levels among the different organs examined. From the microarray data of the IR64 rice cultivar, four of these genes (*OsCam1-1*, *OsCML7*, 9 and *13*), were expressed at more or less constant levels in different developmental stages. However, expression patterns among different organs/tissues that are not consistent with those analyzed from the microarray database may also result from variation in the rice varieties examined.

Up-regulated expression of a gene in response to a stress signal may reflect the function of the corresponding gene product, especially in signal cascades. In a large gene family, investigating expression patterns of their members could point to genes or isoforms that potentially function under the conditions of interest. In this study, the transcript expression analysis of *OsCam1-1* and nine *OsCML* genes in the KDML105 rice cultivar by rt-RT-PCR revealed that expression levels of *OsCam1-1*, *OsCML4*, *5*, *8*, and *11* were increased under osmotic stress (20% (w/v) PEG) (Figure 3B), which is consistent with the GSE6901 data for the IR64 cultivar from the DNA microarray database (Figure 3A) [12] and suggests that these genes may function in the mechanisms of Ca^2+^-mediated responses to osmotic stress. Similarly, expression of these genes was also found to increase in the KDML105 rice cultivar under salt stress (150 mM NaCl) along with *OsCML1* (Figure 3C), suggesting that *OsCam1-1*, *OsCML1*, *4*, *5*, *8,* and *11* may function in the mechanisms of Ca^2+^-mediated responses to salt stress. The up-regulated transcript expression levels under salt stress of *OsCML4*, *8* and *11* were also consistent with the GSE6901 data for the IR64 cultivar from the DNA microarray database (Figure 3A). Whether the discrepancy in the transcript expression patterns of *OsCam1-1*, *OsCML1*, and *5* is due to the different timings of expression being monitored or differences in the rice varieties examined remains to be evaluated but, overall, up-regulation of almost the same set of the highly conserved *OsCML* genes under osmotic and salt stresses was observed, confirming that conclusion that genes responsive to osmotic stress overlap to a high extent with those that are responsive to salt stress [24].

Several of these genes not only exhibited up-regulated transcript expression levels by osmotic stress and salt stress, but also exhibited different patterns of up-regulation in terms of timing and levels of expression within a tissue type and exhibited differential expression in different tissues/organs. Together, differential temporal and spatial expression patterns of these *OsCam1-1* and *OsCML* genes suggest that each individual gene product may possess specific roles during Ca^2+^-mediated responses to osmotic stress and salt stress. Comparison of the transcript expression patterns of these genes under osmotic or salt stress in the KDML105 rice cultivar, as determined by rt-RT-PCR in this study, with the publicly available RNA-Seq data and the other microarray data sets [25], revealed that *OsCML4* and *OsCML8* consistently exhibited higher expression levels under osmotic and salt stresses suggesting their significant functions in Ca^2+^-mediated responses to these stimuli. In addition, the early induction within 1 h of *OsCam1-1* and *OsCML5* under osmotic and salt stresses, and of *OsCML1* under salt stress suggests their importance in conveying the stress signals early in the transduction cascades of Ca^2+^ signaling. However, caution must be taken in interpreting these results because the changes in levels of transcripts generally do not coincide with the changes in the levels of the proteins they encode and, given the time it takes to express a protein, may not necessarily reflect their involvement in the responses of the cell to stress. Nonetheless, a gene of which transcript expression is early induced would be given special attention as a candidate for further investigation into its possible involvement in response to a particular stress.

Several cis-acting elements in response to abiotic stresses in the 5^′^ upstream regions of the *OsCam1-1* and *OsCML* genes were detected (Figure 4). DREs, important promoter elements that are responsive to drought, high salt, and cold [16], were located in the putative promoters of *OsCam1-1*, *OsCML4*, *5*, *7*, and *11*. Consistent with this is that the transcript expression level of all these genes was shown by rt-RT-PCR to increase in the KDML105 rice cultivar under osmotic and salt stresses with *OsCML7* exhibiting a slight increase in its expression level and the others significantly much higher level of up-regulation. DREs specifically interact with the transcription factors DREBs and regulate expression of many stress-inducible genes. In rice, the DRE binding protein 1 (*Os*DREB1) functions in the cold stress response, whereas *Os*DREB2 functions in the heat and osmotic stress responses [26]. The results here indicate that these DREs are potentially responsible for the osmotic stress-induced expression of these genes and interesting candidates for further characterization.

In the 5^′^ upstream region of the *OsCam1-1* promoter, the putative DRE motif (ACCGAC) was located at −1062. Induction of the GUS activity level in the three independent *OsCam*::*gus* transgenic rice plants (Figure 5) suggested that induction of *OsCam1-1* expression under salt stress is, at least, partly due to the activity of its promoter. Its induction appears to be dependent on the severity of the salt stress (concentration of NaCl), with no significant induction in the plants treated with 100 mM NaCl while a monophasic and biphasic induction were observed in the plants treated with 150 mM and 300 mM NaCl, respectively, in all three independent transgenic lines. A biphasic induction of *OsCam1-1* expression by salt stress in the KDML105 rice cultivar, as determined by rt-RT-PCR has been reported previously [27], whilst heat shock (HS) induced biphasic [Ca^2+^_cyt_ signal in rice root cells and the HS-induced expression of *OsCam1-1* strongly oscillated [11]. The complex responses of the *OsCam1-1* gene to salt stress suggest that *OsCam1-1* is a significant player in the Ca^2+^ signal transduction network under salt stress. However, caution must be taken in interpreting these results, especially on timings of the induction because of the possible differences in mRNA and protein stability between the *gus* gene and the *OsCam1-1* gene.

## Conclusions

Differential expression patterns in tissues/organs were reported among different *Cam* and *CML* genes in rice. Under osmotic and salt stresses, expression of several genes including *OsCam1-1*, *OsCML4*, *5*, *8*, and *11* was induced with different kinetics and magnitude. In agreement with their inducibility, several *cis*-acting elements in response to abiotic stresses including DREs, important promoter elements responsive to drought, high salt, and cold, were detected in the 5^′^ upstream regions of these genes. Induction in the GUS activity of the transgenic rice expressing *gus* gene under the control of the *OsCam1-1* promoter, which contains a putative DRE motif (ACCGAC) at −1062 was observed. Its induction appeared to be biphasic and dependent on the severity of salt stress. These results suggest that these *OsCam* and *OsCML* genes play differential roles as sensor relays in regulating developmental processes and Ca^2+^-mediated responses to abiotic stress.

## Methods

### Materials

TRI REAGENT was purchased from Molecular Research Center, Inc. (Cincinnati, OH, USA). The iScript cDNA synthesis kit and the SsoFast EvaGreen Supermix were purchased from Bio-Rad (Hercules, CA, USA). 4-methylumbelliferyl β-D-glucuronide (4-MUG) and 4-methylumbelliferone (4-MU) were from Sigma (St. Louis, MO, USA). Synthetic oligonucleotides for real-time reverse transcription polymerase chain reaction (rt-RT-PCR) were obtained from 1^st^ Base (Singapore). Seeds of *Oryza sativa* L. cultivar Khao Dawk Mali 105 (KDML105) were provided by the Rice Research Center (Patumthani, Thailand).

### Plant growing and stress treatments

*O. sativa* ‘KDML105’ seedlings were hydroponically grown in nutrient solution under a 12-h light/12-h dark photoperiod. After three weeks, the plants were treated with 150 mM NaCl for 1, 3, 6, 12, 24 or 48 h by adding the same medium except supplemented with 150 mM NaCl or 20% (w/v) PEG4000 into the chamber containing the rice seedlings with minimal touch contact and disturbance. Rice seedlings were grown with a completely randomized design (CRD) with three replicates, and for each replicate, ten seedlings were pooled for RNA extraction.

### Real-time reverse transcription polymerase chain reaction (rt-RT-PCR)

Leaf blades/sheaths of three-week old seedlings; or leaf blades/sheaths, roots, flowers and seeds during the grain filling period were collected and immediately frozen in liquid nitrogen and stored at −80°C. Rice tissues were ground in liquid nitrogen to a fine powder using a chilled mortar and pestle. Total RNA from the frozen tissues was extracted using the TRI REAGENT with 0.2 mL chloroform and precipitated by mixing with isopropanol. Subsequently, cDNA was synthesized by RT-PCR using the iScript cDNA synthesis kit according to the manufacturer’s instruction. Then, rt-PCR was performed in a final volume of 20 μL, which contained a 2-μL aliquot of the first strand cDNA reaction, 0.05 mM of each of the gene-specific primers (Table 1), and 1x SsoFast EvaGreen Supermix. The reaction included an initial 8 min at 95°C, followed by 40 cycles of 95°C for 30 s; Ta°C for 30 s and 72°C for 45 s (where the annealing temperature, Ta, was as in Table 1). The specific oligonucleotide primers for the second stage of the rt-RT-PCR (rt-PCR of the cDNA), shown in Table 1, were selected using the Primer3 algorithm [28] from the *OsCam1-1* and *OsCML* cDNA sequences (see sequence analysis below).

The *O.sativa* elongation factor-1α (*OsEF1α*) was assumed to be independent of the abiotic stresses in its expression levels (a housekeeping gene), and so amplified as an internal control using the specific primers OsEF1*α*-F/R (Table 1), designed as above against the corresponding sequence (GenBank accession code AK105030). The level of transcript expression of the *OsCam1-1* and respective *OsCML* genes expression was then standardized against that for the *OsEF1α* gene transcript expression level from the same cDNA template as an internal control, and then expressed with reference to the standardized transcript expression level on day 0 of the treatment. Rt-RT-PCR reactions of each cDNA preparation were performed in tripicate for technical replication. Data were compared using the analysis of variance (ANOVA), and then the means were compared with Duncan’s multiple range test (DMRT) accepting significance at the p < 0.05 level.

### Determination of β-glucuronidase (GUS) activity

The homozygous seeds of three independent *OsCam1-1*:*gus* transgenic rice lines [10] were hydroponically grown in 0.25 × strength NB medium [29] under a 16-h light/8-h dark photoperiod and treated with salt stress by adding, with minimal contact and disturbance, the medium containing 0 (control), 100, 150 or 300 mM NaCl into the chamber containing the rice seedlings to be treated. The leaf tissues of two-week-old rice seedlings were collected and frozen in liquid nitrogen at 0, 1, 2, 4, 8, 24 and 48 h after treatment. Rice seedlings were grown in a CRD with four replicates, and for each replicate, two seedlings were pooled for RNA extraction. The collected tissues were ground with a chilled mortar and a pestle, and homogenized in protein extraction buffer (50 mM Sodium phosphate, pH 7.0, 10 mM EDTA, 10 mM β-Mercaptoethanol, 0.1% Sodium n-lauroylsarcosine, 0.1% Triton X-100). The β-glucuronidase enzyme (GUS) reaction assay was performed in protein extraction buffer containing 1 mM 4-MUG. For each reaction the substrate solution (200 μL) was pre-incubated at 37°C for 5 min, and then the reaction was initiated by the addition of 1 μL of the test plant extract and incubated at 37°C. After 10, 20, 30 and 40 min of incubation time 10 μL aliquots of the reaction mixture was transferred to 100 μL of stop solution (0.2 M Na_2_CO_3_). The standard curve was generated using five concentrations (10 nM to 100 nM) of 4-MU. The fluorescence intensity of the samples was measured by setting the excitation at 365 nm and the emission at 455 nm. The β-glucuronidase activity of the protein extracts was calculated as pmole 4-MU produced per minute per μg protein (units per μg protein). Fluorescence of each replicate was measured three times. Protein concentrations of the samples were determined by the Bradford’s method [30] using bovine serum albumin as a standard. Data were compared using ANOVA, and the means were compared with REGWQ accepting significance at the p < 0.05 level.

### Sequence analysis and microarray data retrieval

To determine which *OsCML* genes may have altered expression patterns in response to stress, their expression levels in different rice organs and tissues were compared. DNA microarray data of the *OsCam* and *OsCML* genes were retrieved from the publicly available microarray data of global gene expression from the experiment GSE 6893, which examined rice gene expression in various rice tissues/organs and stages of reproductive development from the IR64 rice cultivar or GSE 6901, which examined gene expression of 7-day-old ‘IR64’ rice seedlings grown under 3 h of dehydration (drought) or salt stress (200 mM NaCl) [12] via the rice oligonucleotide array database [25] available at ( http://www.ricearray.org/).

For PCR design, nucleotide sequences from *O.sativa* were retrieved from of the Rice Genome Annotation Project database [31] and GenBank at the National Center for Biotechnology Information ( http://www.ncbi.nlm.nih/). Sequences of the following genes (loci): *OsCam1-1* (LOC_Os03g20370), *OsCML1* (LOC_Os01g59530), *OsCML3* (LOC_Os12g03816), *OsCML4* (LOC_Os03g53200), *OsCML5* (LOC_Os12g41110), *OsCML7* (LOC_Os08g02420), *OsCML8* (LOC_Os10g25010), *OsCML9* (LOC_Os05g41200), *OsCML11* (LOC_Os01g32120) and *OsCML13* (LOC_Os07g42660) were obtained.

To identify *cis*-acting regulatory DNA elements within the promoters of the *OsCam1-1* and *OsCML* genes, their 5^′^ upstream sequences were analyzed using a Database of Plant Cis-acting Regulatory DNA Elements (PLACE) ( http://www.dna.affrc.go.jp/PLACE/) and the Plant Promoter Analysis Navigator (PlantPAN) ( http://plantpan.mbc.nctu.edu.tw/).

## Competing interests

The authors declare that they have no competing interests.

## Authors’ contributions

AC carried out the laboratory work along with KL and SP; and prepared figures and tables. KL and TB participated in database searches and data analyses. TL and TB performed data interpretation and drafted the manuscript. All authors read and approved the final manuscript.

## References

[B1] White PJ Broadley MR Calcium in Plants Ann Bot 2003 92 478 511 10.1093/aob/mcg164 PMC4243668 12933363

[B2] McAinsh MR Pittman JK Shaping the calcium signature New Phytol 2009 181 275 294 10.1111/j.1469-8137.2008.02682.x 19121028

[B3] Kretsinger RH Nockolds CE Carp muscle calcium-binding protein J Biol Chem 1973 248 3313 3326 4700463

[B4] Day IS Reddy VS Shad Ali G Reddy ASN Analysis of EF-hand-containing proteins in Arabidopsis Genome Biol 2002 3 research0056.1-0056.24 10.1186/gb-2002-3-10-research0056 PMC134623 12372144

[B5] Boonburapong B Buaboocha T Genome-wide identification and analyses of the rice calmodulin and related potential calcium sensor proteins BMC Plant Biol 2007 7 4 10.1186/1471-2229-7-4 17263873 PMC1797041

[B6] DeFalco TA Bender KW Snedden WA Breaking the code: Ca^2+^ sensors in plant signalling Biochem J 2010 425 27 40 10.1042/BJ20091147 20001960

[B7] Hashimoto K Kudla J Calcium decoding mechanisms in plants Biochimie 2011 93 2054 2059 10.1016/j.biochi.2011.05.019 21658427

[B8] McCormack E Braam J Calmodulins and related potential calcium sensors of Arabidopsis New Phytol 2003 159 585 598 10.1046/j.1469-8137.2003.00845.x 33873603

[B9] Chinpongpanich A Wutipraditkul N Thairat S Buaboocha T Biophysical characterization of calmodulin and calmodulin-like proteins from rice, Oryza sativa L Acta Bioch Bioph Sin 2011 43 867 876 10.1093/abbs/gmr081 21908855

[B10] Phean-o-pas S Limpaseni T Buaboocha T Structure and expression analysis of the OsCam1-1 calmodulin gene from Oryza sativa L BMB Rep 2008 41 771 777 10.5483/BMBRep.2008.41.11.771 19017488

[B11] Wu HC Luo DL Vignols F Jinn TL Heat shock-induced biphasic Ca^2+^ signature and OsCaM1-1 nuclear localization mediate downstream signaling in acquisition of thermotolerance in rice (^Oryza sativa L.^) Plant Cell Environ 2012 10.1111/j.1365-3040.2012.02508.x 22428987

[B12] Jain M Nijhawan A Arora R Agarwal P Ray S Sharma RS Kapoor S Tyagi AK Khurana JP F-box proteins in rice. Genome-wide analysis, classification, temporal and spatial gene expression during panicle and seed development, and regulation by light and abiotic stress Plant Physiol 2007 143 1467 1483 10.1104/pp.106.091900 17293439 PMC1851844

[B13] Prestridge DS SIGNAL SCAN: A computer program that scans DNA sequences for eukaryotic transcriptional elements CABIOS 1991 7 203 206 2059845 10.1093/bioinformatics/7.2.203

[B14] Higo K Ugawa Y Iwamoto M Korenaga T Plant ^cis^-acting regulatory DNA elements (PLACE) database Nucleic Acids Res 1999 27 297 300 10.1093/nar/27.1.297 9847208 PMC148163

[B15] Chang W-C Lee T-Y Huang H-D Huang H-Y Pan R-L PlantPAN: Plant promoter analysis navigator, for identifying combinatorial cis-regulatory elements with distance constraints in plant gene groups BMC Genomics 2008 9 561 574 10.1186/1471-2164-9-561 19036138 PMC2633311

[B16] Dubouzet JG Sakuma Y Ito Y Kasuga M Dubouzet EG Miura S Seki M Shinozaki K Yamaguchi-Shinozaki K *OsDREB* genes in rice, Oryza sativa L., encode transcription activators that function in drought-, high-salt- and cold-responsive gene expression Plant J 2003 33 751 763 10.1046/j.1365-313X.2003.01661.x 12609047

[B17] Fujita Y Fujita M Shinozaki K Yamaguchi-Shinozaki K ABA-mediated transcriptional regulation in response to osmotic stress in plants J Plant Res 2011 124 509 525 10.1007/s10265-011-0412-3 21416314

[B18] Baker SS Wilhelm KS Thomashow MF The 5'-region of ^Arabidopsis thaliana cor15a has cis^-acting elements that confer cold-, drought- and ABA-regulated gene expression Plant Mol Biol 1994 24 701 713 10.1007/BF00029852 8193295

[B19] Heo WD Lee SH Kim MC Kim JC Chung WS Chun HJ Lee KJ Park CY Park HC Choi JY Cho MJ Involvement of specific calmodulin isoforms in salicylic acid-independent activation of plant disease resistance responses Proc Natl Acad Sci USA 1999 96 766 771 10.1073/pnas.96.2.766 9892708 PMC15211

[B20] Van der Luit AH Olivari C Haley A Knight MR Trewavas AJ Distinct calcium signaling pathways regulate calmodulin gene expression in tobacco Plant Physiol 1999 121 705 714 10.1104/pp.121.3.705 10557218 PMC59432

[B21] Duval FD Renard M Jaquinod M Biou V Montrichard F Macherel D Differential expression and functional analysis of three calmodulin isoforms in germinating pea (^Pisum sativum L^.) seeds Plant J 2002 32 481 493 10.1046/j.1365-313X.2002.01409.x 12445120

[B22] Liu H-T Sun D-Y Zhou R-G Ca^2+^ and AtCaM3 are involved in the expression of heat shock protein gene in Arabidopsis Plant Cell Environ 2005 28 1276 1284 10.1111/j.1365-3040.2005.01365.x

[B23] Ishida H Huang H Yamniuk AP Takaya Y Vogel HJ The solution structures of two soybean calmodulin isoforms provide a structural basis for their selective target activation properties J Biol Chem 2008 283 14619 14628 10.1074/jbc.M801398200 18347016

[B24] Zeller G Henz SR Widmer CK Sachsenberg T Rätsch G Weigel D Stress-induced changes in the ^Arabidopsis thaliana transcriptome analyzed^ using whole-genome tiling arrays Plant J 2009 58 1068 1082 10.1111/j.1365-313X.2009.03835.x 19222804

[B25] Jung KH Dardick C Bartley LE Cao P Phetsom J Canlas P Seo YS Shultz M Ouyang S Yuan Q Frank BC Ly E Zheng L Jia Y Hsia AP An K Chou HH Rocke D Lee GC Schnable PS An G Buell CR Ronald PC Refinement of light-responsive transcript lists using rice oligonucleotide arrays: evaluation of gene-redundancy PLoS One 2008 3 e3337 10.1371/journal.pone.0003337 18836531 PMC2556097

[B26] Mizoi J Shinozaki K Yamaguchi-Shinozaki K AP2/ERF family transcription factors in plant abiotic stress responses Biochim Biophys Acta 2011 1819 86 96 21867785 10.1016/j.bbagrm.2011.08.004

[B27] Saeng-ngam S Takpirom W Buaboocha T Chadchawan S The role of the OsCam1-1 salt stress sensor in ABA accumulation and salt tolerance in rice J Plant Biol 2012 55 198 208 10.1007/s12374-011-0154-8

[B28] Rozen S Skaletsky HJ Krawetz S, Misener S Primer3 on the WWW for general users and for biologist programmers. In Bioinformatics Methods and Protocols: Methods in Molecular Biology 2000 Humana Press, New Jersey 365 386 10.1385/1-59259-192-2:365 10547847

[B29] Li L Ou R de Kochkc A Fauquet C Beachy RN An improved rice transformation system using the biolistic method Plant Cell Rep 1993 12 250 255 10.1007/BF00237129 24197151

[B30] Bradford MM A rapid and sensitive method for the quantitation of microgram quantity of protein utilizing the principle of protein dye binding Anal Biochem 1976 72 248 254 10.1016/0003-2697(76)90527-3 942051

[B31] Ouyang S Zhu W Hamilton J Lin H Campbell M Childs K Thibaud-Nissen F Malek RL Lee Y Zheng L Orvis J Haas B Wortman J Buell RC The TIGR Rice Genome Annotation Resource: improvements and new features Nucleic Acids Res 2007 35 D883 887 Database issue 10.1093/nar/gkl976 17145706 PMC1751532

